# Chronic cerebral aspects of long COVID, post‐stroke syndromes and similar states share their pathogenesis and perispinal etanercept treatment logic

**DOI:** 10.1002/prp2.926

**Published:** 2022-02-16

**Authors:** Ian Albert Clark

**Affiliations:** ^1^ Research School of Biology Australian National University Canberra ACT Australia

**Keywords:** chronicity of neurological deficit, etanercept, fatigue, long COVID, post‐stroke syndromes, taste and smell, TNF

## Abstract

The chronic neurological aspects of traumatic brain injury, post‐stroke syndromes, long COVID‐19, persistent Lyme disease, and influenza encephalopathy having close pathophysiological parallels that warrant being investigated in an integrated manner. A mechanism, common to all, for this persistence of the range of symptoms common to these conditions is described. While TNF maintains cerebral homeostasis, its excessive production through either pathogen‐associated molecular patterns or damage‐associated molecular patterns activity associates with the persistence of the symptoms common across both infectious and non‐infectious conditions. The case is made that this shared chronicity arises from a positive feedback loop causing the persistence of the activation of microglia by the TNF that these cells generate. Lowering this excess TNF is the logical way to reducing this persistent, TNF‐maintained, microglial activation. While too large to negotiate the blood‐brain barrier effectively, the specific anti‐TNF biological, etanercept, shows promise when administered by the perispinal route, which allows it to bypass this obstruction.

## Abstract 2

This article is about how the neurological aspects of long COVID (fatigue, neurogenic pain, loss of ability to taste or smell), can be best understood, and treated. For this purpose, the literature on TNF and brain function, both physiological and disease‐associated, and how this is being applied to various long‐term neurodegenerative conditions, is discussed.

## Graphical abstract

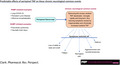

**Abbreviations**
AMPA: α‐amino‐3‐hydroxy‐5‐methyl‐4‐isoxazolepropionic acid
BBB: blood‐brain barrier
DAMP: damage‐associated molecular patterns
HMGB1: high‐mobility group box 1
PAMP: pathogen‐associated molecular patterns
POCD: post‐operative cognitive deficit
PTSD: post‐traumatic stress disorder
rTNF: recombinant TNF
TBI: traumatic brain injury
TLRs: Toll‐like receptors

## 1 BACKGROUND

Having been a party to the literature on the cytokine concept of disease pathogenesis since its inception^1^ has generated a strong awareness in this author that the concept has its doubters. Certainly this can be unsettling for those to whom the minimalist explanations for post‐stroke syndromes and traumatic brain disease (TBI) have, in their terms, served them well clinically. For example, the dogma in understanding and dealing with ischemic stroke – treating intravenously within a few hours with intravenous tissue plasminogen activator (tPA) to break up the blood clot, in order to minimize the degree of damage – still dominates treatment of this condition. Indeed, most stroke research still concerns how to make tPI treatment more effective, since it is predicated on rapid treatment after the stroke being crucial because ischemic brain cells soon die, and cannot be resurrected. A corollary has been that nothing can be done about the syndromes observed in post‐stroke patients, who, permanently deprived of the neurons that died, can expect to have limited function for the rest of their lives. Similarly, pathogens are often deemed to kill cells directly. Thus teaching and learning about infectious diseases runs the risk of being largely limited to the specific diagnosis of the pathogen in order to select the anti‐pathogen agent with the best contemporary reputation.

Despite the advent of awareness of pathogen‐associated molecular patterns (PAMPs) and damage (or danger) associated molecular patterns (DAMPs), and the activities of the cytokines thus induced through Toll‐like, and functionally similar pattern recognition receptors in increased concentrations (discussed below), these earlier mindsets can also dominate research into both these non‐infectious and infectious conditions, preventing them being discussed together, using a common knowledge base. As the title infers, this commentary is intended to foster this cytokine approach becoming widespread enough to allow a broader understanding of shared features of what are, traditionally, seen as profoundly different illnesses, with very different causes. In particular, discussion focusses on how a number of chronic neurological changes can be common to certain conditions, be they infectious or non‐infectious.

## 2 HOW PAMPS AND DAMPS LINK THE PATHOGENESIS OF INFECTIOUS AND NON‐INFECTIOUS DISEASE

It seems appropriate to expand here on the above PAMP and DAMP terminology, since it is central to understanding the pathophysiology of both infectious and non‐infectious diseases, including their influence on neurological function. Further detail of this concept, attributable to Janeway^2^ and Matzinger,^3^ has been reviewed before from our group, albeit in an Alzheimer's disease context.^4^ These insights illuminate a useful concept for explaining how not only molecular patterns on pathogens (grouped as PAMPs), but also patterns revealed by host tissue damage or hypoxia (grouped as DAMPs), act to release TNF and related cytokines. In brief, they do so by these molecular patterns recognizing sites such as Toll‐like receptors (TLRs) expressed on or in many cells, but most strongly on macrophages and microglia, for which they act as agonists. This terminology facilitates explaining the natural history of two infectious diseases, one bacterial (Lyme disease) one viral (COVID‐19), both triggered by PAMPs, in the same terms as non‐infectious states such as post‐stroke syndrome and TBI triggered by DAMPs, as is done here. Despite this difference in what initiates them, the illness they cause can often be very similar, with diverse and complex cerebral symptoms persisting after the pathogen in the case of Lyme disease, or the acute hypoxia in the case of stroke, or tissue damage in TBI, have long gone.

Clinical similarities within acute infectious diseases can also be rationalized within this framework. At the Dana‐Faber Institute in Boston, when rTNF was being tested for killing tumors in patients,^5^ and before the PAMP/DAMP concept had been elucidated, these authors and I exchanged views on the side effects thus generated. To these researchers this toxicity mimicked influenza, whereas it reminded me more of severe malaria. Two other groups have since independently reported influenza mimicry by rTNF administration.^6^, ^7^ Years later this striking diagnostic overlap became official in a blinded trial conducted by clinical experts in influenza and falciparum malaria.^8^ This is strikingly consistent with two different PAMPs, one on a virus, the other on a protozoan, generating the one illness by inducing TNF, an activity for which they have both been on record for some time. It also rationalizes why the syndromes seen in acute COVID‐19, bacterial sepsis, and Lyme disease all have influenza high on their differential diagnosis list.

## 3 HMGB1 AS AN EXAMPLE OF A BROADLY SYNERGISTIC DAMP

To illustrate the DAMP principle more broadly, the wide spectrum of involvement of high‐mobility group box 1 (HMGB1) warrants brief discussion. This DNA‐binding protein is evolutionarily conserved (e.g. is present in shellfish^9^ as well as humans). Indeed, it is constitutively expressed within the nucleus of virtually all types of cells. As a nuclear protein released from damaged cells or those undergoing non‐lethal physiological stress^10^ and secreted by activated leukocytes, HMGB1 is a common intermediary between cellular stress, or physical damage, and TNF release. Moreover, hypoxia,^11^ and TNF itself,^12^ can induce HMGB1 release. Thus it synergizes with both DAMPs and PAMPs.^13^ HMGB1 is reported to function as such not only during the pathogenesis of Lyme disease,^14^ but also COVID‐19,^15^ influenza,^16^ TBI,^17^, ^18^ and the cognitive loss that can follow post‐operative cognitive deficiency (POCD).^19^ Ischemia also induces HMGB1,^20^ with its importance implied by the ability of an anti‐HMGB1 antibody to improve brain dysfunction in a rat model of cardiac arrest.^21^ Thus HMGB1 illustrates how closely interwoven are the signaling pathways that drive disease pathogenesis in both infectious and non‐infectious states.

## 4 TNF IN BIOLOGY AND DISEASE

The capacity to generate the cytokine TNF evidently appeared extremely early in biological evolution, and has been scrupulously retained. Remarkably, the human TNF molecule, or at least its receptor‐binding portion, pre‐dates bilateral symmetry, in that *Acropora* spp., the major reef‐builder corals, generate a TNF whose receptor recognizes human TNF.^22^ It is not surprising that a molecule so rigorously preserved has proven to be widely and essentially involved in physiology^23^ and disease^24^ of more complex creatures such as insects and fish, as well as the physiology and disease in all vertebrates so far examined. It also has roles in mediating innate immunity. Most TNF is generated by macrophages stimulated by PAMPs or DAMPs, with microglia, the cerebral equivalent of macrophages, taking over the role within the blood‐brain barrier. Reducing its excess levels in chronic non‐cerebral inflammatory diseases such as rheumatic arthritis, Crohn’s disease and psoriasis has proved to be an enormous clinical success, but its application in neurological conditions is as yet in its infancy. This is partly because its physiological roles in the brain are so subtle and complex, but commercial sparring within a highly competitive field also plays a large role in preventing this being broadly appreciated.

## 5 PHYSIOLOGICAL ROLES OF TNF IN THE CENTRAL NERVOUS SYSTEM

TNF has an astonishing number of essential roles in normal brain tissue. This is reviewed in some detail here in order to demonstrate how dependent normal brain function is the widespread homeostatic roles of this cytokine, for example through controlling neuronal plasticity.^25^ TNF and other members of the TNF superfamily of cytokines^26^ mediate neurite outgrowth, normal fetal development of nociception, and the survivability, excitability and cell differentiation mediated by nerve growth factor.^27^ Its biological influence spans generations, with a requirement for adequate maternal TNF to induce, in milk, the chemokines needed for normal hippocampal development and memory in offspring.^28^ TNF released during physiological neuronal activity plays a crucial role in regulating the strength of normal synaptic transmission.^29^ Moreover, there has been evidence for some time now that TNF governs behavioral phenotypes in physiological ageing, without immunological challenge.^30^

As we have reviewed,^31^ free synaptic glutamate, which is central to synaptic function, is largely regulated by TNF's control over both glutaminase and certain key glutamate re‐uptake transporters. Thus TNF, one of the few cytokines styled as gliotransmitters, has, as reviewed,^32^ subtle but effective control over synaptic physiology, influencing AMPA receptors on synapses, synaptic plasticity (considered, by Hebbian theory, to be an important foundation of memory and learning), and long‐term potentiation, a paradigm for how memory may be consolidated at the molecular level. In excess it can lead to glutamate excitotoxicity, which is discussed later. In other words, the brain requires low levels of properly orchestrated TNF for normal physiological function. Clearly this level has to fluctuate as physiology requires. Normal physiological neuronal activity therefore requires TNF to be released in homeostatically controlled quantities from microglia, astrocytes and neurons before it is cleared by TNF receptors.

TNF is also involved in normal neurotransmission via modulating excitatory inputs,^32^ trafficking of AMPA receptors,^33^ homeostatic synaptic scaling,^34^ and long‐term potentiation.^35^ Furthermore, it maintains normal background levels of neurogenesis.^36^ Mitochondrial function depends on TNF,^37^ as does regulation of the neurotransmitter, orexin,^38^ which, as we recently reviewed,^39^ controls sleep, motor control, focused effort, appetite and water intake. TNF also regulates neuronal type‐1 inositol trisphosphate receptors (IP3R), which are central to neuronal Ca^++^ homeostasis, and thus the ionic signaling cascades on which normal function of neurons depends.^40^ This large functional overlap between the brain and the innate immune system described in the infectious disease literature is reinforced in a comprehensive review by others.^41^ Through this bigger picture, the subtle but widespread functional changes detected by administering the then newest revisions of the WAIS‐III and WMS‐III mental performance tests to chronic Lyme disease patients^42^ can be better appreciated. Equally, it is small wonder that a recent study of a relatively young cohort of COVID‐19 survivors reported that a substantial proportion exhibited cognitive dysfunction.^43^

Clearly, all the above functions are vulnerable to TNF being outside its physiological range when it is over‐induced by either PAMPs or DAMPs that can enter, or be generated in, the brain. The capacity of microglia, via the cytokines these cells generate, to influence neuronal function, are regularly reviewed.^44^ Thus, increased cerebral levels of microglial‐origin TNF, whether arising from induction by PAMPs on pathogens such as SARS‐CoV‐2, influenza viruses or *Borrelia* spp., by DAMPs (e.g. post‐stroke syndromes) or, as discussed below, non‐infectious influences such as hypoxia or trauma, can alter brain function in the same way.^45^ Understanding the physiological roles of TNF in biology gives us the opportunity to visualize how the incredibly wide range of subtle function loss reported during persistent Lyme disease,^46^ long COVID‐19,^47^ and post‐stroke neurological changes, might arise. This arguably bewildering range and degree of changes is, nevertheless, consistent with what can seem, at first sight, an equally bewildering array of pleiotropic functions of TNF in neurophysiology, as outlined above. This degree of complexity is entirely compatible with the subtle intellectual and memory deficits documented to affect many performance tasks in persistent Lyme disease.^42^ It is telling of that period that these authors should have inserted the caveat that further work was needed to definitively rule out non‐specific illness effects on cerebral function. Nowadays, in contrast, we can infer from the literature in this and the above paragraph that little if anything specific is involved in persistent Lyme disease. By rationalizing this non‐specificity, this approach may well shed new light on settling the controversies that have plagued our understanding of this disease for decades.

Once TNF, IL‐1β, IL‐6, IL‐4 and other host‐origin cytokines were identified, interacting pathways proposed, and laboratory reagents available, an awareness of their roles in states such as infectious and inflammatory disease, sickness behavior, ageing, and neurodegeneration dominated their literatures. Consequently, the infectious disease world often demonstrates little awareness that, at lower concentrations, these cytokines have many essential roles in brain physiology. Homeostatic normal synaptic plasticity and scaling as well as associated glutamate control, neurogenesis, neuromodulation, synaptic function, memory,^29^ learning,^48^ and cognitive function^49^ are particularly important examples. We discussed these aspects of TNF further in 2010,^50^ and similar roles of other cytokines have been updated since.^41^ Importantly, all of this low‐level fluctuating cerebral cytokine activity is also necessary for homeostasis in healthy brains. As summarized earlier, such homeostasis depends on these cytokines being appropriately and autonomously orchestrated, both in precise locations and minute concentrations, within the bounds of neurophysiology.

## 6 HOW CEREBRAL MICROGLIAL ACTIVATION AND TNF GENERATION CAN PERSIST

The next challenge is to understand why such neurophysiological change can be rendered chronic, whether in post‐stroke syndromes, Lyme disease, COVID‐19, and influenza encephalopathy survivors, whereas systemic cytokine levels and associated acute symptoms do not persist. In 2007 it was reported that TNF generation in the mouse, after intraperitoneal injection of bacterial LPS, persists in the CSF for very much longer (at least 10 months) than in the serum (6 h).^51^ It is plausible, therefore, that the microglia whose cytokines flow into this CSF do not become LPS‐tolerant, as do mouse macrophages after exposure to LPS.^52^ Human macrophages also prove to react to LPS in this way.^53^

These intriguing sets of observations and proposals are consistent with the report^54^ of a positive feedback loop in the activation of microglia by the TNF that these microglia generate. In other words, an autocrine loop exists whereby TNF can prolong the activation of the cerebral microglia that generate it. Others have since invoked an intermediary role for brain‐derived neurotropic factor (BDNF) in this feedback microglial activation.^55^ These cellular interactions have been further investigated from another angle by Puntener and co‐workers in Southampton.^56^ In brief, they concluded that the innate immune cells in the brain do not become tolerant to systemic infection, but are primed instead. In their words, these studies are consistent with prolonged and damaging cytokine production that may have a profound effect on the onset and/or progression of pre‐existing neurodegenerative disease. Others interested in the same challenge^57^ have, by tracking cerebral single‐cell gene‐expression profiles in long COVID, observed a strong increase across key inflammatory pathways in what they term as the choroid‐to‐cortex network. Thus, they propose, inflammation is relayed into the brain parenchyma. This group also noted that microglial and astrocyte subpopulations associated with COVID‐19 share features seen in human neurodegenerative disease. These same concepts warrant examination in persistent Lyme disease, as well as in brains from post‐stroke and post‐TBI syndrome models.

A precedent for the existence of long‐term microglial activation, with a DAMP rather than a PAMP the original cause, has been reported in ischemic stroke,^58^ subarachnoid hemorrhage,^59^ and TBI.^60^, ^61^ Since this was demonstrated *in vivo* in two of these three quoted studies, it seems pressing for the techniques in the previous paragraph to be applied to influenza, persistent Lyme disease and long COVID brains. This concept is also consistent with the endotoxin tolerance being observed systemically but not in the brain. Importantly, it implies that perispinally administered etanercept (discussed later) can deactivate these microglia by inactivating this excess TNF, hence removing the positive feedback loop that causes its production to persist. In this way these PAMP and DAMP‐induced cytokines, and not the pathogen, ischemia or trauma directly, are argued here to be the essential cause of most of the commonly seen neurological dysfunction in the persistent stage of all these illnesses. Logically, therefore, this principle applies to the persistent cerebral phases of COVID‐19, influenza or Lyme disease as well as to encephalopathies seen post‐stroke or in TBI survivors.

## 7 INFECTIOUS AND NON‐INFECTIOUS CONDITIONS SHARING THE SAME POST‐ACUTE CEREBRAL SEQUELAE

### 7.1 Influenza encephalopathy survivors

Having already covered similar ground with long COVID,^62^ here we discuss the wider literature that has accumulated with influenza encephalopathy. A convenient point to start recounting it is the work of Jurgens and co‐authors, who reported that a mouse model exhibited increased microglial activity and increased inflammatory cytokine generation in the hippocampus, and impaired reversal learning in the Morris water maze.^63^ Others^64^ subsequently confirmed these cytokine and microglial observations, as well as an accompanying deficit in spatial learning. Importantly, others^65^ have recently discussed the concept of post‐acute sequelae caused by non‐persistent viruses causing chronic inflammation.

### 7.2 Stroke, traumatic brain injury and cardiac arrest survivors

Since these are non‐infectious conditions, their pathophysiology is exclusively DAMP‐driven, mostly by hypoxia and the products of tissue damage. Non‐infectious inflammatory conditions such as post‐stroke syndromes^66^ provide a convenient way to demonstrate the importance of TNF, however it is generated, in conditions that share this clinical picture. Thirty years ago Ghezzi and co‐workers reported that hypoxia – the basis of cerebral stroke – greatly increases TNF generation by human monocytes, which are closely related to microglia.^67^ Consequences include mitochondrial DNA and HMGB1 escaping from damaged mitochondria^68^ and cell nuclei.^69^ For example, persistent HMGB1 release has been confirmed in patients who have recently experienced stroke,^70^ TBI,^18^ and cardiac arrest.^71^ The cytokines thereby secreted by these cerebrally‐released DAMPs inevitably generate the same characteristic group of symptoms as seen in long COVID‐19, persistent Lyme disease, and influenza encephalopathy. Examples are given in the next paragraph.

## 8 THE RANGE OF CHRONIC CEREBRAL SYMPTOMS IN WHICH EXCESS TNF IS INCRIMINATED

**FIGURE 1:**
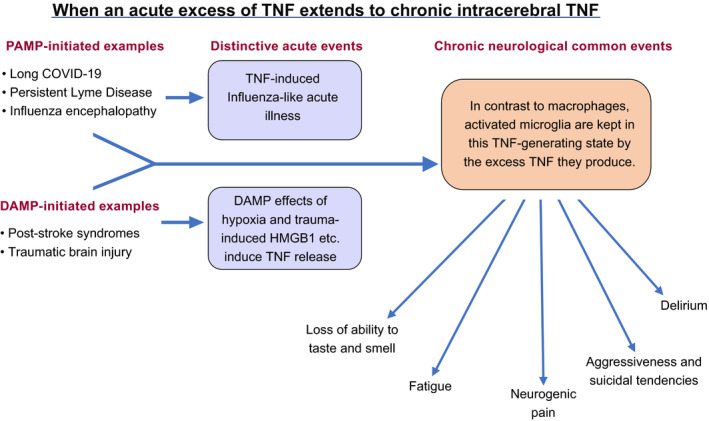
When an acute excess of TNF extends to chronic intracerebral TNF An illustration of how a range of chronic external stimuli that present pathogen‐associated molecular patterns or pathogen‐associated molecular patterns to various Toll‐like receptors on different cerebral cellular components, and thus induce TNF and TNF‐induced cytokines. In contrast to activated macrophages, activated microglia maintain their TNF‐generating state, generating a series of distinctive clinical consequences

A cluster of chronic symptoms form the syndrome observed when cerebral TNF becomes inappropriately excessive for the normal function of a particular cerebral site. Below, we discuss fatigue, neurogenic pain, delirium, aggressiveness, and suicide tendency as examples. The ingredients in a particular mix can be expected to depend on the site and pattern of excessive induction of TNF, independent of whether the driving force is a PAMP or a DAMP (Figure 1). Even within a single syndrome, further differences might well also result from different genetic backgrounds or epigenetic modifications in individuals. In short, from a pathogenesis perspective these symptoms are all under the one TNF umbrella, whether infectious or non‐infectious in origin. Thus there is a rationale for excessive cerebral TNF, and thus the same chronic symptoms, to persist, either when a pathogen is no longer present (e.g. long COVID and persistent Lyme disease), or is an irrelevancy (e.g. post‐stroke syndromes and TBI).

### 8.1 Loss of ability to taste and smell

Two of the clinical states in which loss of capacity to appreciate taste or smell are commonly reported, post‐stroke and long COVID, are discussed throughout this text. One of this pair being infectious, and the other not, nicely fits the argument made throughout that the pathogenesis of the syndromes discussed here is independent of whether their inducer was a PAMP or a DAMP. Moreover, the literature explains loss of taste, taste distortion^72^ and smell^73^ in mice, and at least taste in humans,^74^ in excess TNF terms. Within a series of 629 consecutive case reports of post‐stroke syndromes, treatment with perispinal etanercept reportedly restored long‐term loss of capacity to taste and smell.^75^ It will be interesting to observe the outcome when these two measures are included as end points in one of the series of post‐stroke random control trials presently being conducted,^76^ as well as when this aspect of long COVID is formally investigated.

### 8.2 Fatigue

Fatigue is an undisputed and regularly documented component of long COVID, persistent Lyme disease and viral infections as diverse as influenza and a range of arbovirus infections. As with delirium and aggression, the presence of fatigue fits the pattern of the later stages of these infectious conditions sharing symptoms with non‐infectious neurodegenerative states such as post‐TBI^77^ and post‐stroke.^78^

Considering that the original evidence that TNF would induce fatigue was inadvertent, it is very impressive indeed. In 1988 open trials comprising 115 treatments in 50 patients, recombinant TNF (rTNF) was infused over 24 h in the hope of causing tumor regression. Quite unexpectedly, this induced fatigue so severe that hospital discharge was considerably delayed.^5^ Independently, Steinmetz reported marked fatigue in all of 16 tumor patients given a single 24 h infusion of rTNF at a range of doses. Indeed, the main dose‐limiting effect was termed profound prostration.^6^ Nearly 20 years later, Cavadini and co‐workers^79^ reported that TNF suppressed the activity of the PAR bZip clock‐controlled genes *Dbp*, *Tef*, *Hlf*, and the period genes *Per1*, *Per2 and Per3*, all of which are involved in controlling circadian rhythm. They reasoned that this suppression, through influencing sleep cycles, provides a causal link between TNF and the fatigue of disease. In 2012, we summarized the implications of these changes in clock gene function for mitochondrial dysfunction, and thus reduced systemic and cerebral energy availability.^80^ The case was subsequently made^81^ for excess TNF diminishing the intracellular movement of mitochondria required for optimal ATP generation, leading to their disintegration and a resultant diminished capacity of the organism to utilize oxygen.^82^ The chronically activated microglia that can be expected to be the source of this chronic excess TNF – see two Sections back, and in Figure 1 – have already been documented in chronic fatigue patients.^83^ Case reports of successfully treating post‐stroke fatigue with perispinally administered etanercept are published.^75^ Decrease in fatigue is one of the primary outcomes being measured in a current post‐stroke perispinal random controlled trial, as were other parameters in the first.^76^

### 8.3 Neurogenic pain

Neurogenic pain, a term encompassing both neuropathic (nerve damage) and central (CNS‐origin) pain, is commonly seen in both long COVID‐19 disease^84^ and persistent Lyme disease,^85^ as well as post‐stroke syndromes.^86^ For some time evidence has been accumulating that excess levels of cytokines such TNF, and IL‐1β downstream from it, are responsible.^75^, ^87^, ^88^, ^89^ The most recent of this study series^89^ used an animal model of the perispinal route used by Tobinick and co‐workers that employed a fluorophore‐labelled TNF antibody to track it. Following a decade of case report experience, the first random controlled trial of perispinal etanercept in stroke patients^76^ successfully included alleviation of neurogenic pain as an endpoint (see^90^ for an invited Commentary article).

As observed in other vector‐spread viral diseases, such as Chikungunya and Dengue, persistent Lyme disease can include arthralgia. Typically this does not respond readily to antibiotics, implying it is not directly caused by the pathogen.^91^ In a mouse model designed to respond to the observation that the degree of pain that patients experience does not correlate with quantitative measures of joint inflammation,^92^ Lopes and co‐workers^93^ recently studied experimental pain that persists after inflammation has resolved. Through intracerebroventricular injection of anti‐TNF antibodies, they established that this pain is neurogenic in origin. Hence the Lyme arthritis recently discussed by Coiffier and colleagues,^94^ and many others previously, is plausibly neurogenic, involving TNF‐mediated pain.

### 8.4 Delirium

Delirium is variously described as an acute and profound disturbance of thinking, memory, orientation, perception and emotion. Transient delirium is common in intensive care units, and has been perceptively described as an extreme manifestation of the sickness behavior caused by TNF and the other cytokines it induces.^95^ Although described in Lyme disease,^96^ COVID‐19,^97^, ^98^ cerebral malaria,^99^ and influenza,^100^ delirium is just as much at home in the literature on non‐infectious encephalopathies. Examples are post‐ stroke syndromes,^101^ TBI,^102^ and post‐operative cognitive deficit (POCD),^103^ where PAMPs are absent, but the cytokines PAMPs induce – in this case induced by DAMPs – are plentiful. POCD has been reported to be prevented by prior treatment with dexmedetomidine,^104^ an agent that, as has been reviewed,^105^ possesses anti‐TNF properties. For example, dexmedetomidine inhibits induction of this cytokine by unmethylated CpG DNA, a strong DAMP, and a model for the DAMP activity of other unmethylated DNA such as that of bacterial or mitochondrial origin.^106^

### 8.5 Aggressiveness and suicidal tendencies

Behavioral changes, such as aggressiveness, are also recorded in persistent Lyme disease.^107^ As with delirium, aggressiveness has also been observed in the persistent neurological impairments after cerebral malaria in African children. The syndrome often includes persistent deficits in cognition, learning ability at school, attention, memory, visuo‐spatial and language skills that often begin after recovery from acute cerebral malaria.^108^ These changes, reported to last for at least 9 years, are apparently a consequence of prior cerebral malaria which, in terms of parasite clearance, has been successfully treated. Memorably, the literature includes reports of development of unprovoked aggression towards peers and throwing stones at people and cars with no or minimal provocation, as well as uncontrollable anger.^108^ Such persistent neurological impairments after cerebral malaria are evidently not uncommon, having been recently discussed as imposing an unbearable burden on the neuro‐rehabilitation services of Malawi.^109^ CSF levels of TNF have been shown to be increased in the chronic neurological impairment of falciparum malaria.^110^ Again as with delirium, aggressiveness does not require an infectious agent to initiate the type and degree of encephalopathy required for its manifestation. Two non‐infectious examples are post‐stroke^111^ and post‐TBI.^77^

The relevant literature on suicidal tendencies, plausibly termed self‐aggression in this context, also warrants summarizing here. Risk of suicide is reportedly increased in many of the conditions being discussed here, both infectious (Lyme disease^112^ and long COVID‐19^113^) and non‐infectious states with tissue damage and consequent DAMP release (TBI^114^ and post‐traumatic stress disorder (PTSD)). The latter is widely accepted to be a tragedy of a particular magnitude for returned service personnel.^115^ It is well‐recognized that pro‐inflammatory cytokines such as TNF are chronically raised in PTSD.^116^ The nature of this subsection of this review also draws attention to the literature that focusses on TNF in order to gain an understanding of mechanisms of psychiatric conditions.^117^, ^118^

Therefore, should cerebral TNF, the initiator of the above cytokine cascade, and mostly generated in the brain by microglia, become inappropriately increased through the advent of an infectious disease such as COVID‐19^119^ or Lyme disease,^120^ we can expect that mental health, including memory, cognition, and alertness, would be harmed. Circulating cytokine levels can inform about the immune response and the acute disease, but tell us little about why the neurological symptoms seen in the chronic stage of these acute infections occur. Importantly, these post‐acute sequelae also can be said to occur in non‐infectious disease states where the same cytokines are generated by DAMPs. In these circumstances, such as in post‐stroke and TBI syndromes, the acute stage consisted of trauma (TBI) or ischemia (post‐stroke syndromes), and pathogens are clearly irrelevant. In other words, chronically increased intracerebral cytokines causes these chronic changes in which pathogens play no part. This is consistent with the argument that antibiotics are inappropriate treatments for persistent Lyme disease.^85^

## 9 THERAPEUTIC IMPLICATIONS

Above, the case is made that the neuropathophysiology that brings about the symptoms of post‐stroke syndromes, TBI, long COVID‐19, persistent influenza and Lyme disease are essentially identical. This may seem to be counterintuitive, since the first two have non‐infectious origins, while the other three are initiated by infectious agents. As we have indicated, their commonality arises because DAMPs and PAMPs both activate the same intracerebral cytokine TNF‐driven pathways. These regulatory pathways are essential in the homeostatically managed low, appropriately fluctuating TNF concentrations required to maintain brain health, but when over‐stimulated they throw neurophysiology out of kilter. Inevitably, the harmful changes that ensue are common across the board, irrespective of whether they were initiated by DAMPs (as with hypoxia or trauma) or PAMPs (as with virus, bacteria or protozoan).

As discussed earlier, changes in cerebral function, often subtle, but sometimes disabling, can become persistent. Inactivating chronically activated microglia by removing the excess TNF that maintains this state^54^ is a logical explanation for this fundamental and rapid reversal to normal seen in post‐stroke case studies^75^ and the one random control trial to date.^76^

As referenced earlier in the present text, post‐stroke syndromes, as well as other non‐infectious neurodegenerative diseases, typically exhibit the same fatigue, neurogenic pain, delirium and aggressiveness as are recorded in persistent Lyme and long COVID‐19. This is consistent with the chronic cerebral TNF generation that maintains microglia in a chronically activated state, rather than the initial pathogen, being a plausible therapeutic target in persistent Lyme disease. Gabapentin, generally regarded as an anticonvulsant, is a synthetic analogue of gamma aminobutyric acid. Hampered by dose‐limiting side effects, it has nevertheless been used, with some success, against chronic neurogenic pain, including in Lyme disease.^121^ It has been demonstrated to reduce TNF levels,^122^ arguably because of its capacity to enhance IL‐10, a recognized inhibitor of TNF generation.^123^

## 10 CONTROLLING GLUTAMATE CYTOKINE TOXICITY

Glutamate is a key physiological excitatory neurotransmitter in virtually all activities of the nervous system, yet in excess it is extremely harmful. As we expanded upon 5 years ago^31^ this combination of functional importance and potentially high toxicity demands tight control over its release and re‐uptake. Crucially for understanding the outcome of etanercept therapy, both glutamate release^124^, ^125^ and subsequent re‐uptake^126^ are inhibited by excessive cerebral TNF.

Various alphaviruses cause the same chronic neurological sequelae as seen in COVID‐19 and influenza. The glutamine analogue 6‐diazo‐5‐oxo‐L‐norleucine (DON), albeit too toxic to be a practical drug, is a useful experimental agent. As does TNF,^124^ DON blocks the conversion of glutamine to glutamate by inhibiting glutaminase,^127^ thus decreasing the likelihood of cerebral glutamate excitotoxicity. DON has been used to ameliorate this aspect of alphavirus infection.^128^ As the authors note, these studies indicate that neuroprotection with agents that decrease inflammation and excitotoxic damage is an encouraging approach to preventing these sequelae. Others^129^ have used etanercept in a TBI model, noting attenuation of TBI‐induced increased cerebral cellular levels of glutamate and the lactate‐to‐pyruvate ratio, as well as return of motor and cognitive function. We have previously summarized^31^ the two mechanisms whereby excess TNF contributes to glutamate excitotoxicity. Given the capacity of excess TNF to simultaneously increase synaptic glutamate production by activating glutaminase, while also inhibiting the glutamate re‐uptake proteins, it very effectively enhances glutamate accumulation, and thus toxicity, at synapses. Being a glutamine analogue, DON is unlikely to influence the glutamate clearance step. This implies that specific anti‐TNF agents, such as etanercept, can be expected to be more effective than DON, which has only one string to its bow, at treating this condition.

## 11 PERISPINAL ETANERCEPT, AND THE RATIONALE FOR ITS USE

With the realization that TNF is as central to normal signaling and pathophysiology in the central nervous system, as we have reviewed,^50^ the therapeutic challenge, because of its molecular size, has been to get a specific anti‐TNF biological through, or around, the blood‐brain barrier in pharmaceutical amounts. Intriguingly, after being turned down by one of the pair of etanercept patent holders in 2005, and despite the skepticism of these Pharmas and their collaborating academic neurologists,^130^ an independent practitioner/researcher in another medical specialty appears to had indeed found a way to do so. Specifically, he had developed a novel route of administering etanercept for this purpose, as outlined below.

The perispinal route consists of a shallow injection of the dose into the cerebrospinal venous system.^131^ This is followed by a head‐down tilt for 5 min^132^, ^133^ on the grounds that this allows, through reverse flow, rapid entry of large molecules into the CSF through the valveless veins that usually drain the cerebrospinal fluid.^134^, ^135^ Hence the blood‐brain barrier is bypassed, rather than penetrated. The history of appreciating the details of this drainage is well‐documented,^136^ and has recently been summarized by others.^137^ Clearly, the pattern, rapidity, and distribution of clinical effects best fit with rapid delivery of etanercept via retrograde distribution into the cerebral venous system, the choroid plexus, and the cerebrospinal fluid from the external vertebral venous division of Batson’s plexus. Developing this approach involved two freely accessible steps: understanding the implications of the relevant anatomy and physiology^134^, ^135^, ^138^ and appreciating the gist of an aviation medicine text.^139^ While investigating the effects of negative G force generated by head‐down stress on the integrity of the blood brain barrier (BBB), these researchers tilted anaesthetized rabbits head‐down for varying periods. After 5 min of tilting the concentration of albumin, normally outside the BBB, was dramatically enhanced in the CSF. Taking note of the implications of this work, Tobinick subsequently demonstrated the *in vivo* brain distribution of etanercept, a molecule with a size comparable to albumin, after perispinal injection in a rat model.^140^

**FIGURE 2:**
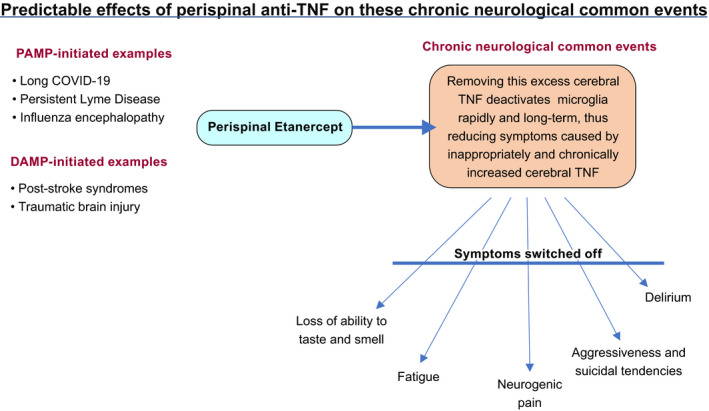
Predictable effects of perispinal anti‐TNF biologicals on these chronic neurological common events The distinctive clinical consequences of chronic cerebral excessive production. These symptoms can be predicted to dissipate once cerebral TNF levels are restored to its normal homeostatic levels [Correction added on 24 February 2022 after first online publication: The word 'TNF' has been corrected to 'anti‐TNF' in this current version of figure 2.]

Therefore treatment with perispinally administered etanercept, plus five minutes tilting head‐down (<45 degrees, in practice), can be predicted, from literature discussed earlier, to de‐activate the chronically TNF‐generating microglia, kept activated by chronically increased cerebral TNF.^54^ This can be expected to switch off chronic TNF production by these microglia, and explain the permanent restoration of much cerebral normality in case studies^75^, ^141^ and the initial RCT^76^ (Figure 2). The specific intention is to remove, permanently, the chronic symptoms that can be expected to persist indefinitely, long after the acute effects of PAMPS and DAMPs have passed. It has already been proposed as an argument for reversing the neurological aspects of long COVID‐19^62^ and post‐cerebral malaria cognitive impairment.^142^

The initial observation that perispinal treatment with a specific anti‐TNF biological typically causes such a rapid return to normal neurological function was as striking as it was unexpected across the field. Certainly, large players can find the phenomenon more convenient to dismiss than to attempt to understand. My invited 2017 editorial to comment on a Clinical Advisory issued by the American Academy of Neurology (AAN)^130^ discusses an example of this. This startling rapidity of full and durable return of function surely, to this writer, points to homeostasis being restored to a normal signaling function of TNF, as discussed earlier. The alternative hypotheses are removing the potential involvement of TNF by suppressing pathogens via innate immunity^65^ or removing its ability to kill neurons.^143^ Neither are at all consistent with the observed rapid response.

Attention is drawn to the curious phenomenon of the same encephalopathies being often defined only in TNF or IL‐1β terms. Whatever their origin, infectious and non‐infectious encephalopathies are, beyond the nature of the initial inducer, very similar in pathogenesis. Yet certain encephalopathy publications^144^ give center stage to IL‐1β, mentioning TNF only in passing. Other have the opposite emphasis, depending, typically, on what patents their funder holds. In reality, once the initial PAMP or DAMP event arising from a viral or bacterial infection (PAMP‐driven), or from trauma or hypoxia (DAMP driven), has occurred, the microglia begin to generate a stock range of cytokines. Some of these, such as IL‐1β, will inevitably be present whether a PAMP or DAMPs induces the TNF, which has activated the caspase‐1 that converts pre‐ IL‐1 to IL‐1β, a process for which indirect evidence was first reported in decades ago.^145^ Subsequently, in an *in vitro* cerebral context, exposure of a human neuroblastoma cell line to TNF was shown to promote oxygen radical‐mediated caspase‐1 activation and thence IL‐1β secretion.^146^ This was largely the basis of developing the inflammasome concept.

Not surprisingly, therefore, anti‐TNF inhibits IL‐1 generation.^147^, ^148^ Moreover, infliximab, the first of the clinical anti‐TNF biological agents, has been reported to reduce functionally similar cytokines, for example IL‐1 as well as TNF levels.^149^, ^150^, ^151^ It also reduces IL‐6^148,150^ and IL‐8.^150^ Hence every time TNF is mentioned in this text one can infer that IL‐1β is a downstream fellow traveler and collaborator. Indeed in the publication, forty years ago, the first to argue for such cytokines causing disease as well as innate immunity, we assayed for IL‐1 – then termed lymphocyte activating factor (LAF) – in addition to TNF, and discussed them together.^1^ In the same decade, before it was appreciated that one induced the other, Carl Nathan catalogued their impressively similar activities documented by the late 1980’s.^152^ For the purposes of this text we have given the TNF perspective because this is the focus of most of the relevant literature. This may of course be biased by much wider availability of rTNF and specific anti‐TNF biologicals than their anti‐IL‐1β counterparts. Even so, etanercept and similar biologicals can be predicted to be useful in conditions in which IL‐1β dominates the literature.

In conclusion, it is obvious from the literature on the pathogenesis and therapy of all of the conditions discussed here, systemic and cerebral, infectious and non‐infectious, that they are yet to be considered in an integrated manner. Largely, silos still reign. Aspects of the depth of this need for productive cross‐fertilization are cognitively expressed in a recent commentary with a more clinical perspective.^153^ Unfortunately these authors did not stray into the realm of neurologists.

## CONFLICTS OF INTEREST

The author declares no conflict of interest or financial involvement in the research discussed here.

## Data Availability

No data available in the study.
