# Corrigendum

**DOI:** 10.1002/prp2.942

**Published:** 2022-03-04

In Clark (2022)^1^, in Figure 2, the word “TNF” has been updated as “anti‐TNF.”

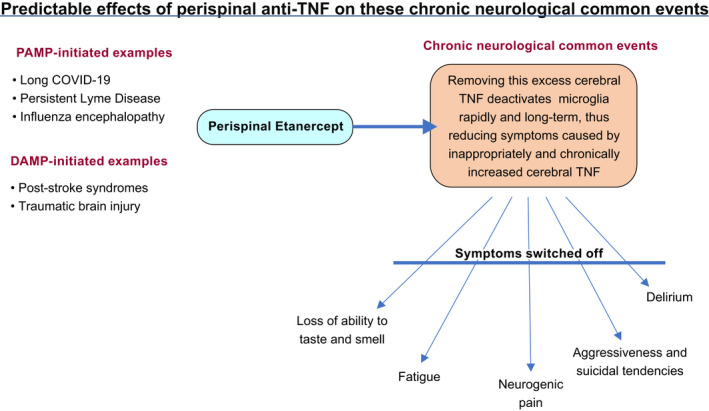

The updated Figure 2 is shown below:

The online version has been updated to reflect this change.
